# Multiple sclerosis: early indicators of disease and assessing future risk

**DOI:** 10.1007/s00415-020-10239-1

**Published:** 2020-10-08

**Authors:** M. D. Willis, N. P. Robertson

**Affiliations:** 1 Department of Neurology, University Hospital Wales, Cardiff, CF14 4XN UK; 2 Institute of Psychological Medicine and Clinical Neuroscience, Cardiff University, University Hospital of Wales, Heath Park, Cardiff, CF14 4XN UK

Over the last decade, the number of disease-modifying treatments (DMTs) available to patients with multiple sclerosis (MS) has exponentially increased in number. For clinicians equipped with this armamentarium, who and when to treat to maximise therapeutic efficacy, whilst being ever mindful of potential serious side effects has become a focus of routine clinical practice.

With the severity of the MS clinical phenotype ranging so widely from benign to aggressive disease, the ability of a clinician to be able to make an accurate forecast of a patient’s disease trajectory is of great value. Stratifying patients based on available clinical and radiological data allows an informed decision to be made about the intensity of disease surveillance and the balance of risk and benefits of different therapeutic agents. However, this is an imprecise science and further studies aiding understanding of this are to be welcomed. Once a diagnosis is made, early treatment with immunosuppressive or immunomodulating therapies is increasingly favoured to prevent future episodes of neuroinflammation and disability accumulation in the longer term. As such, any novel methods to detect and diagnose disease at an earlier stage are an area of keen interest to neurologists working in this field.

In this months’ journal club, we consider three papers whose aim is to aid earlier diagnosis and predict disease development and trajectory. First, Outteryck and colleagues investigate the use of optical coherence tomography in aiding the detection of both symptomatic and asymptomatic optic nerve lesions in patients with clinically isolated syndrome. This technique may have clinical utility where optimal radiological imaging is unavailable for the detection of optic nerve lesions and potentially in the development of future MS diagnostic criteria. In the second paper, Lebrun-Frenay et al. evaluate the 10-year risk of a clinical event following a diagnosis of radiologically isolated syndrome (RIS). This large cohort study is informative for both clinicians and patients alike in understanding future development of disease. In the third paper, the MSBase study group identify early clinical markers of aggressive MS development and reveal easily accessible parameters to help identify such patients. These studies will help clinicians in evaluating the risk of MS disease development and subsequent disease progression. In turn, this will inform predictions of disease trajectory, aid patient counselling and inform individual management decisions.

## Optical coherence tomography for detection of asymptomatic optic nerve lesions in clinically isolated syndrome

Optic nerve lesions are commonly encountered as part of the clinically isolated syndrome (CIS). Despite this, they are not considered as part of the criteria to identify dissemination in space (DIS). Inclusion in these criteria may be of potential clinical utility in identifying DIS at an earlier stage. Optical coherence tomography (OCT)—and in particular, the intereye retinal thickness difference (IETD)—is a potentially sensitive modality to study optic nerve lesions. This study aimed to evaluate the diagnostic accuracy of the IETD in a cohort of patients with CIS for both symptomatic and asymptomatic optic nerve lesions.

One hundred and thirty patients with a CIS in the previous 2.5–4.5 months were prospectively recruited. Demographic and clinical data were collected with each patient undergoing a 3 T MRI brain scan (3D-DIR sequence) and OCT on the same day. For each optic nerve, the presence or absence of DIR hypersignal was recorded. All patients had typical MS lesions outside of the optic nerve. According to the 2017 McDonald criteria, characteristics for DIS were fulfilled for 117 patients and dissemination in time (DIT) for 112. In total, 105 patients fulfilled MS diagnostic criteria. A clinical episode of unilateral ON occurred in 37 patients and bilateral ON in 2 patients. Two groups of patients were subsequently defined: patients with ON (CIS-ON) and without ON (CIS-NON).

In the CIS-ON group (*n* = 39), imaging demonstrated DIR hypersignal in all 41 symptomatic optic nerves and in 11 asymptomatic optic nerves. Imaging of the CIS-NON group (*n* = 91) demonstrated unilateral optic nerve lesions in 22 patients and bilateral optic nerve hypersignal in 7 patients. Retinal thickness/volume was significantly lower in eyes with symptomatic lesions vs. asymptomatic lesions or vs. eyes without optic nerve lesions. Similarly, eyes with asymptomatic optic nerve lesions had lower retinal thickness/volume than eyes without optic nerve lesions. CIS-ON patients had greater IETD difference compared to CIS-NON patients. For the detection of symptomatic and asymptomatic optic nerve lesions, ganglion cell layer coupled to the inner plexiform layer (GC-IPL) IETD had better performance as compared to peripapillary retinal nerve fiber layer (pRNFL) IETD.

*Comment: *This study confirmed a high frequency of asymptomatic optic nerve lesions and that GC-IPL IETD was the most accurate measure in detecting both asymptomatic and symptomatic lesions. Thresholds for identifying optic nerve lesions were lower than previously reported. The results of this study suggest that IETD may be a potential biomarker for identifying optic nerve lesions. This may aid future longitudinal studies, evaluating whether inclusion of optic nerve lesions in the next iteration of the diagnostic criteria is warranted. This test may also have clinical utility where sensitive MRI detection of optic nerve lesions is unavailable. However, as this study was performed on a single OCT machine and clinical cohort (CIS), the results may not be applicable to all OCT machines and other MS disease states.

Outteryck et al. (2020) Neurology 95(6):e733–e744. https://doi.org/10.1212/WNL.0000000000009832 (Epub 2020 Jul 28)

## Radiologically isolated syndrome: 10-year risk estimate of a clinical event

Radiologically isolated syndrome (RIS) describes brain MRI findings incidentally fulfilling the criteria for dissemination in space (DIS), but without any clinical correlate of inflammatory disease. Although important to recognise through accurate radiological reporting and detailed history taking, the long-term risk of developing a clinical event is unknown and there are no guidelines for surveillance or treatment. This study group (who have previously reported on the 5-year risk) therefore aimed to estimate the risk of a clinical event (either an acute demyelinating event or 12 month in symptom progression) at 10 years. In addition, the association of this risk with baseline demographic, clinical and radiological characteristics was investigated.

This study included the 451 patients from the authors’ original 2014 cohort. All baseline scans fulfilled the 2009 criteria for RIS (2005 criteria for DIS). Demographics, family history, clinical data, neurological examination results, brain and cord MRI findings and CSF were collected at baseline. Patients were followed longitudinally with standardised clinical assessments and MRI performed as per clinical care. The current study obtained data on events that occurred subsequent to 2014—these data were used where available (277 patients, 61.4%), otherwise original data were used.

Of the 451 patients, 86.5% were female with a mean age at RIS diagnosis of 37.2 years and median follow-up time of 6.7 years (range 1–26.6 years). A large proportion of patients were treated with disease-modifying therapy (DMT): non-updated patients, 18.2%; updated patients, 16.7%. Of the total cohort, 173 patients developed symptoms, 21 of which were deemed to be progressive. With regard to the total cohort, the cumulative probability of a first clinical event after 10 years following a diagnosis of RIS was 51.2%. When assessed as prognostic factors, age < 37 years, positive CSF (oligoclonal bands or high IgG index), spinal cord and infratentorial lesions were all independently associated with an increased 10-year risk to first clinical event. When assessed together, these four factors had a cumulative increase in risk with the presence of all four risk factors conveying an 87% risk. In addition, contrast enhancement on follow-up MRI was also significantly associated in the univariate analysis.

*Comment:* This large cohort study with long follow-up estimates the 10-year risk from RIS to first clinical event at 51.2% overall. Four key risk factors were identified—younger age, a positive CSF profile, the presence of spinal cord or infratentorial lesions, and the occurrence of contrast enhancing lesions during follow-up. These results will inform clinicians and help guide discussions with patients. The authors, however, acknowledge a few limitations of this study such as the retrospective design, the variability in MRI scanners, the lack of an adjudication committee for clinical events, and incomplete follow-up for some patients. A high proportion of patients were also exposed to DMTs, which may have affected outcomes.

Lebrun-Frenay et al. (2020) Ann Neurol. https://doi.org/10.1002/ana.25799. (Online ahead of print)

## Early clinical markers of aggressive multiple sclerosis

With a wide spectrum of MS disease activity, the identification of early clinical indicators for disease severity warrants close investigation to help guide surveillance and management. In particular, the clinical predictors of aggressive multiple sclerosis—whereby disability is accrued at an accelerated rate—are not well understood. This study, therefore, aimed to identify early clinical markers, present within the first year of disease, which would indicate an aggressive disease trajectory.

Patients were included in the study if the following criteria were met: (1) a diagnosis of clinically definite relapse-onset multiple sclerosis; (2) age at onset ≥ 18 years; (3) first EDSS recorded within 12 months of symptom onset; (4) at least two recorded EDSS scores within 10 years of symptom onset; and (5) at least 10 years of observation time. Patients were classified as having aggressive multiple sclerosis if they met all three of the following criteria: (1) EDSS ≥ 6 within 10 years of symptom onset; (2) EDSS ≥ 6 confirmed and sustained over ≥ 6 months; and (3) EDSS ≥ 6 sustained until the end of follow-up (≥ 10 years).

Clinical predictors included patient variables (sex, age at onset, baseline EDSS, disease duration at first visit) and recorded relapses in the first 12 months since disease onset (count, pyramidal signs, bowel–bladder symptoms, cerebellar signs, incomplete relapse recovery, steroid administration, hospitalization). For the initial analysis, patient data were obtained from the established international MSBase cohort and Bayesian methods, including Bayesian model averaging (BMA) were used to estimate the risk of aggressive disease in individual patients. Following initial analysis, validation was performed in a second independent cohort (Swedish Multiple Sclerosis Registry).

From the MSBase cohort, 2403 patients met inclusion criteria. Of these, 145 (6%) met criteria for aggressive disease. The mean time from disease onset to aggressive disease definition was 6.05 years. The strongest predictors for aggressive disease included older age at symptom onset (> 35 years) along with high EDSS (≥ 3) and the presence of pyramidal signs in the first year of disease. When evaluated in the second independent cohort, the combination of these three signs was also predictive of aggressive disease. Conversely, the absence of these signs was associated with a lower risk of following an aggressive disease course.

*Comment: *This study identified clinically accessible markers that when identified in the first year since symptom onset suggest an increased risk of an aggressive disease course. These results are of clinical importance, particularly for the identification of patients that will likely benefit from early, aggressive disease-modifying therapy. A limitation of the study included the relative lack of MRI and CSF data. The authors helpfully surmise that early clinical markers of aggressive disease are (1) median EDSS ≥ 3 (2) any pyramidal signs on examination in the first year, and (3) age > 35 at symptom onset.

Malpas et al. (2020) Brain 143(5):1400–1413. https://doi.org/10.1093/brain/awaa081

